# Bitter taste receptors of the zebra finch (*Taeniopygia guttata*)

**DOI:** 10.3389/fphys.2023.1233711

**Published:** 2023-10-04

**Authors:** Praveen Kumar, Ulrike Redel, Tatjana Lang, Sigrun I. Korsching, Maik Behrens

**Affiliations:** ^1^ Leibniz Institute for Food Systems Biology at the Technical University of Munich, Freising, Germany; ^2^ German Institute of Human Nutrition Potsdam-Rehbruecke, Nuthetal, Germany; ^3^ Institute of Genetics, University at Cologne, Cologne, Germany

**Keywords:** bitter taste receptor, calcium mobilization assay, avian, food selection, chemosensation

## Abstract

Despite the important role of bitter taste for the rejection of potentially harmful food sources, birds have long been suspected to exhibit inferior bitter tasting abilities. Although more recent reports on the bitter recognition spectra of several bird species have cast doubt about the validity of this assumption, the bitter taste of avian species is still an understudied field. Previously, we reported the bitter activation profiles of three zebra finch receptors Tas2r5, -r6, and –r7, which represent orthologs of a single chicken bitter taste receptor, Tas2r1. In order to get a better understanding of the bitter tasting capabilities of zebra finches, we selected another Tas2r gene of this species that is similar to another chicken Tas2r. Using functional calcium mobilization experiments, we screened zebra finch Tas2r1 with 72 bitter compounds and observed responses for 7 substances. Interestingly, all but one of the newly identified bitter agonists were different from those previously identified for Tas2r5, -r6, and –r7 suggesting that the newly investigated receptor fills important gaps in the zebra finch bitter recognition profile. The most potent bitter agonist found in our study is cucurbitacin I, a highly toxic natural bitter substance. We conclude that zebra finch exhibits an exquisitely developed bitter taste with pronounced cucurbitacin I sensitivity suggesting a prominent ecological role of this compound for zebra finch.

## 1 Introduction

The sense of taste in animals is required to guide food selection towards nutritive and non-toxic food items (Behrens and Meyerhof, 2018). In general, the vertebrate taste system is equipped with receptive proteins for sweet, salty, umami (the taste of L-amino acids, in case of human mostly L-Glu), sour and bitter (Chaudhari and Roper, 2021). Sour and salty tastes are mediated by the ion channels otopetrin-1 (Tu et al., 2018; Teng et al., 2019; Zhang et al., 2019) and, as a likely candidate sensor, ENaC [(Chandrashekar et al., 2010), but *cf*. (Bigiani, 2020; Lossow et al., 2020; Vandenbeuch and Kinnamon, 2020)]. The remaining taste modalities, sweet, umami and bitter are transduced by G protein-coupled receptors (GPCR) of the taste 1 receptor and taste 2 receptor families [gene symbols: TAS1R1-3 in human, Tas1r1-3 in mouse, other species frequently T1R; TAS2R (number), Tas2r (number), T2R (number)] (Behrens and Meyerhof, 2016). The three taste 1 receptor genes form 2 different heteromers constituting the sweet taste receptor, which consists of TAS1R2 and TAS1R3 subunits and the umami taste receptors with a TAS1R1/TAS1R3 composition (Hoon et al., 1999; Max et al., 2001; Montmayeur et al., 2001; Nelson et al., 2001; Li et al., 2002; Nelson et al., 2002). The bitter taste receptors constitute the taste 2 receptor family, which differs grossly in size between species, ranging from 0 to more than 100 (Behrens and Meyerhof, 2018). The various taste receptor genes are expressed in the oral cavity in specialized cells, which are combined to taste buds (Chaudhari and Roper, 2021). Apart from the oral cavity, taste receptor gene expression has been reported in numerous non-gustatory tissues such as the gastrointestinal tract and the respiratory system (Wang et al., 2020; Martens et al., 2021; Behrens and Lang, 2022). Although in general five basic taste modalities act in concert to assess the nutritional quality of food, a considerable number of animals have lost some taste modalities (Antinucci and Risso, 2017). A well known example is the pseudogenization of the sweet taste receptor in all bird species, although several bird clades with a high demand for sweet tasting nutritional resources such as nectar, achieved the subsequent modification of the umami taste receptor for sweet compound detection (Baldwin et al., 2014; Toda et al., 2021; Cockburn et al., 2022). In the past, it was believed that birds possess an inferior sense of taste, an assumption that was supported by the loss of the sweet tasting ability as well as a small number of bitter taste receptors (Niknafs and Roura, 2018). However, the demonstration of re-gained detection of sweet substances (Baldwin et al., 2014), the finding that the bitter taste receptor repertoires of birds are not generally small as some birds possess a number of intact bitter taste receptor genes matching that of mammals (Davis et al., 2010) and the demonstration that even small bitter taste receptor repertoires can compensate their low number by extraordinary tuning breadths (Behrens et al., 2014), has caused some re-thinking. The first demonstration of profound bitter tasting capabilities in birds was achieved by the functional expression and bitter compound profiling of chicken, turkey and zebra finch bitter taste receptors (Behrens et al., 2014). Despite the low number of only three functional chicken bitter taste receptors, about half of all bitter compounds known to activate human TAS2Rs are detected by the three chicken bitter taste receptors, since each of them responds to a large array of bitter compounds. Similarly, the 2 turkey receptors were found to be broadly tuned, suggesting that a low number of bitter taste receptors alone is not an indication for inferior bitter taste. The same study investigated of in total seven zebra finch Tas2rs the functional properties of three zebra finch Tas2rs that are relatively recent paralogs of chicken Tas2r1. It turned out that the 3 zebra finch receptors each recognize a smaller number of bitter substances compared to the single chicken Tas2r, the Tas2r1, and in fact all three zebra finch receptors together detect as many bitter compounds as the single chicken Tas2r1 (Behrens et al., 2014). Thus, an expanded Tas2r repertoire may allow the development of more specialized receptors. To investigate if the lower tuning breadth of zebra finch Tas2rs might represent a general feature for birds with an elevated number of putatively intact bitter taste receptor genes or if this observation is only true for rather recently evolved paralogs, we screened another zebra finch receptor from a phylogenetically distant branch, the zebra finch Tas2r1. Next, we compared our screening results with chicken, turkey and other zebra finch receptors to assess similarities and differences.

## 2 Materials and methods

### 2.1 Chemicals

Absinthin and Parthenolide were available from previous studies (Brockhoff et al., 2007). Other reagents were purchased as follows: Amarogentin from ChromaDex; Limonin from Apin Chemical; Quassin from CPS Chemie and all other bitter tastants from Sigma-Aldrich.

### 2.2 Database mining and construction of the Tas2r phylogenetic tree

Database mining has been described in a previous study (Behrens et al., 2014). The phylogenetic tree was generated by the Maximum-Likelihood method. The amino acid sequences of the 7 zebra finch bitter taste receptors, the 3 chicken Tas2rs, the 2 turkey Tas2rs, and a turtle (*Pelodiscus sinensis*) bitter taste receptor serving as outgroup were aligned using MAFFT version 7.

### 2.3 Cloning of zebra finch Tas2r cDNA

The cDNA of zebra finch tgTas2r1 was synthesized by MWG Operon and subcloned into vector pcDNA5FRT (Invitrogen). Sequence analysis to verify the integrity of constructs was done by double stranded Sanger sequencing (MWG Eurofins).

### 2.4 Immunocytochemistry

The immunocytochemical staining experiment was mainly done as published previously (Behrens et al., 2021; Ziegler and Behrens, 2021). Briefly, HEK 293T-Gα16gust44 cells grown on poly-D-lysine-coated glass cover slips were transiently transfected with expression constructs coding for zebra finch Tas2r1, -r5, -r6, and –r7. For a negative control, empty expression vector was transfected. On the next day, cells were washed with 37°C warm PBS and fixated with icecold methanol and acetone (1:1, v/v). After washing with PBS at room temperature and blocking buffer (PBS, 5% normal horse serum, 0.5% Triton X-100) treatment, mouse anti-HSV was added at a dilution of 1:15,000 in modified blocking buffer (PBS, 5% normal horse serum, 0.2% Triton X-100). Following thorough rinses with PBS at room temperature, anti-mouse Alexa Fluor488 diluted 1:2,000 in modified blocking buffer was applied for 1 h to facilitate detection of the C-terminally added hsv-epitope. After additional rinses with PBS, cellular nuclei were stained with DAPI, cells were rinsed again with PBS and, finally deionized H_2_O and embedded in Dako mounting medium. Confocal laser scanning microscopy (Zeiss LSM 780) was used to obtain images.

### 2.5 Screening of bitter compounds

The construct of Tas2r1 was transiently transfected in HEK 293T cells stably expressing the G protein chimera Gα16gust44 (Ueda et al., 2003) using Lipofectamine 2000 (Invitrogen) according to the manufacturer’s protocol. HEK 293T-Gα16gust44 cells were cultured in DMEM supplemented with 10% fetal calf serum (FCS) and glutamine. For cultivation of transfected cells as well as functional calcium imaging analyses, the protocols were described previously (Behrens et al., 2021; Lang et al., 2022a; Lang et al., 2022b). The chemicals used for the screening, which was performed between 2014 and 2015, were selected based on chemical diversity from a compound library of substances known to taste bitter to humans. Almost all bitter compounds that were previously tested on bird and frog receptors (41 of 46) (Behrens et al., 2014) were included and expanded by a set of additional compounds. Two different dilutions of test substances in C1-buffer were applied to the cells. Firstly, the highest applicable compound concentration (based on solubility and/or the highest concentration not leading to receptor-independent calcium signals) used previously (Meyerhof et al., 2010; Lossow et al., 2016). Secondly, a 10-fold lower concentration. Calcium signals of Tas2r1 transfected cells and empty vector (negative control) transfected cells were recorded and compared.

### 2.6 Recording and calculations

Cells were seeded, transfected, and stimulated as described for the screening procedure. For the determination of the dose-response relationship of tgTas2r1 with cucurbitacin I, the averaged signal amplitudes were plotted against the logarithm of the compound concentrations. Calculation and plotting was done using SigmaPlot software as published before (Behrens et al., 2021; Lang et al., 2022a; Lang et al., 2022b).

## 3 Results

**FIGURE 1:**
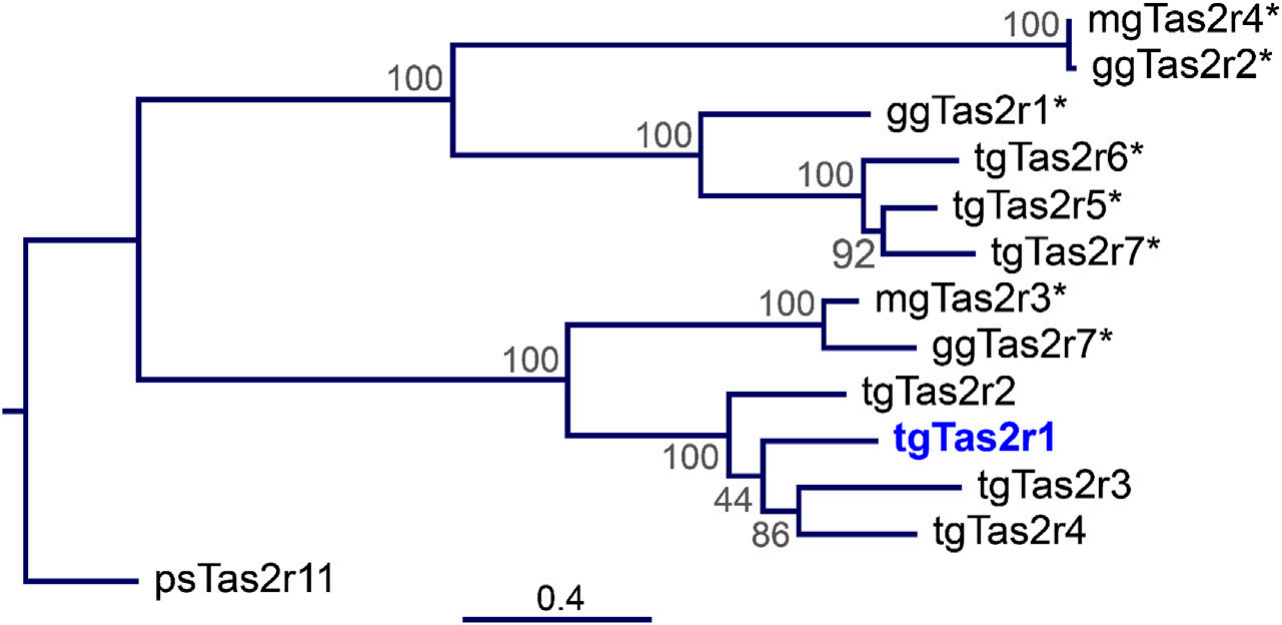
Phylogenetic tree of zebra finch, chicken, and turkey Tas2r. The tree was generated by the Maximum-Likelihood method. The amino acid sequences of the 7 zebra finch bitter taste receptors (tgTas2r1-7), the 3 chicken Tas2rs (ggTas2r1, -r2, -r7), the 2 turkey Tas2rs (mgTas2r3 and –r4), and a turtle bitter taste receptor serving as outgroup (Tas2r11 from *Pelodiscus sinensis*) were aligned using MAFFT version 7. The scale bar at the bottom indicates the phylogenetic distance of peptide sequences. The node numbers represent branch support. Blue, Tas2r1 deorphaned here; asterisks, previously deorphaned Tas2rs.

In contrast to the previously investigated 3 zebra finch tgTas2rs, tgTas2r5, -r6, and –r7, which share a clade with chicken ggTas2r1, zebra finch receptor tgTas2r1 does not belong to these closely related receptor clusters (Figure 1). Instead, tgTas2r1 is a representative of a separate clade, which may be the ortholog of ggTas2r7. This makes this receptor highly interesting for functional studies.

**FIGURE 2:**
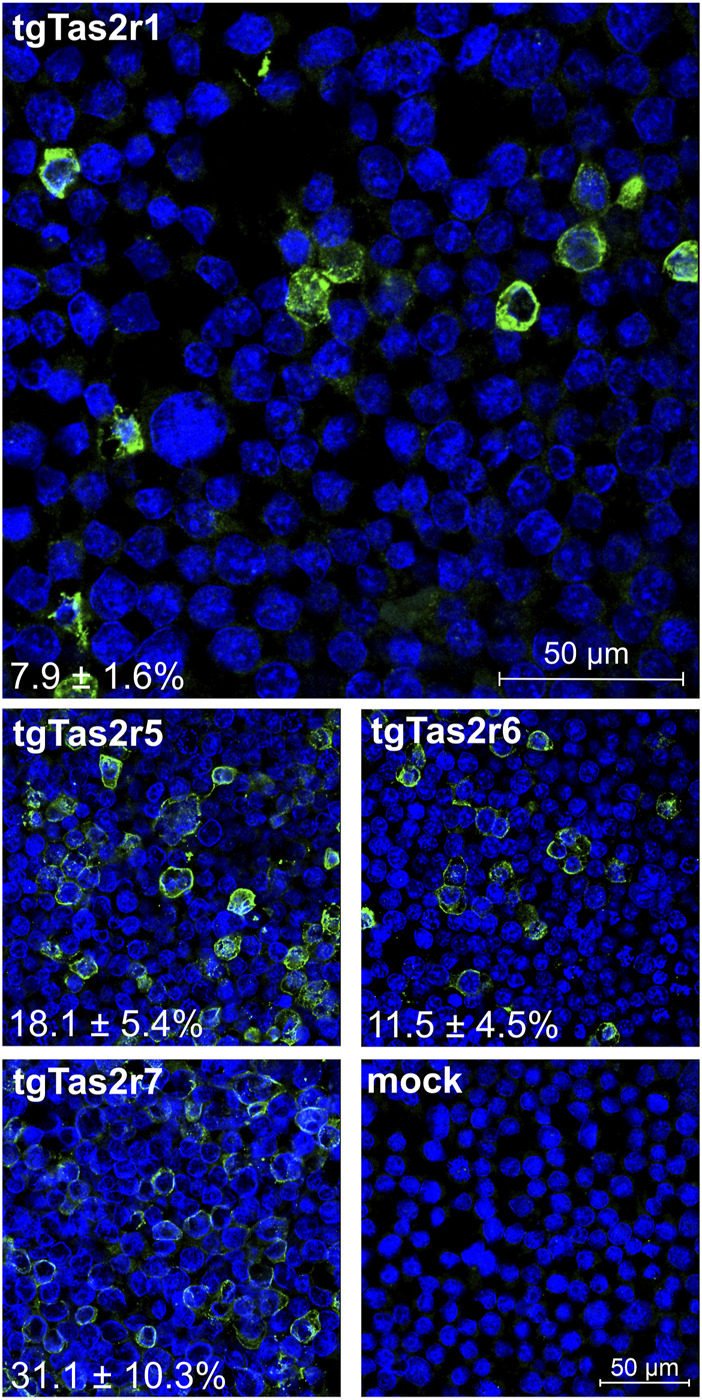
Immunocytochemistry of tgTas2r expression after transient transfection. Expression of tgTas2r1 in Gα16gust44 cells was monitored by confocal laser scanning microscopy using an antiserum against the hsv-epitope attached to the receptor C-terminus (green) and DAPI to visualize nuclei (blue). For comparison also expression constructs coding for tgTas2r5, -r6, and –r7 were analyzed. The specificity of receptor detection is demonstrated by the lack of green signals in identically treated empty vector transfected cells (mock). The constructs used for transient transfection and the determined expression rates (in % ± SD) are labeled in the corresponding panels. The average expression rates were based on the counting of 479–521 cells (nuclei) from 3 representative images by 3 independent persons. Scale bars are shown in the upper and bottom right panels.

To confirm the expression of tgTas2r1, we performed immunocytochemical staining experiments to visualize epitope-tagged receptor proteins along with cell nuclei. For comparisons, we included constructs coding for the previously characterized zebra finch receptors tgTas2r5, -r6, and –r7 (Behrens et al., 2014) and as a negative control empty vector (=mock) transfected cells (Figure 2).

The immunocytochemical experiment confirmed the successful expression of tgTas2r1 in HEK 293T-Gα16gust44 cells. The apparent expression rate reached by transient transfection with tgTas2r1 is 7.9% ± 1.6%, which is lower than the rates observed for tgTas2r5, -r6, and –r7. Nevertheless, the clear detection of receptor tgTas2r1 in the cell line allowed the functional screening as the next step.

**FIGURE 3:**
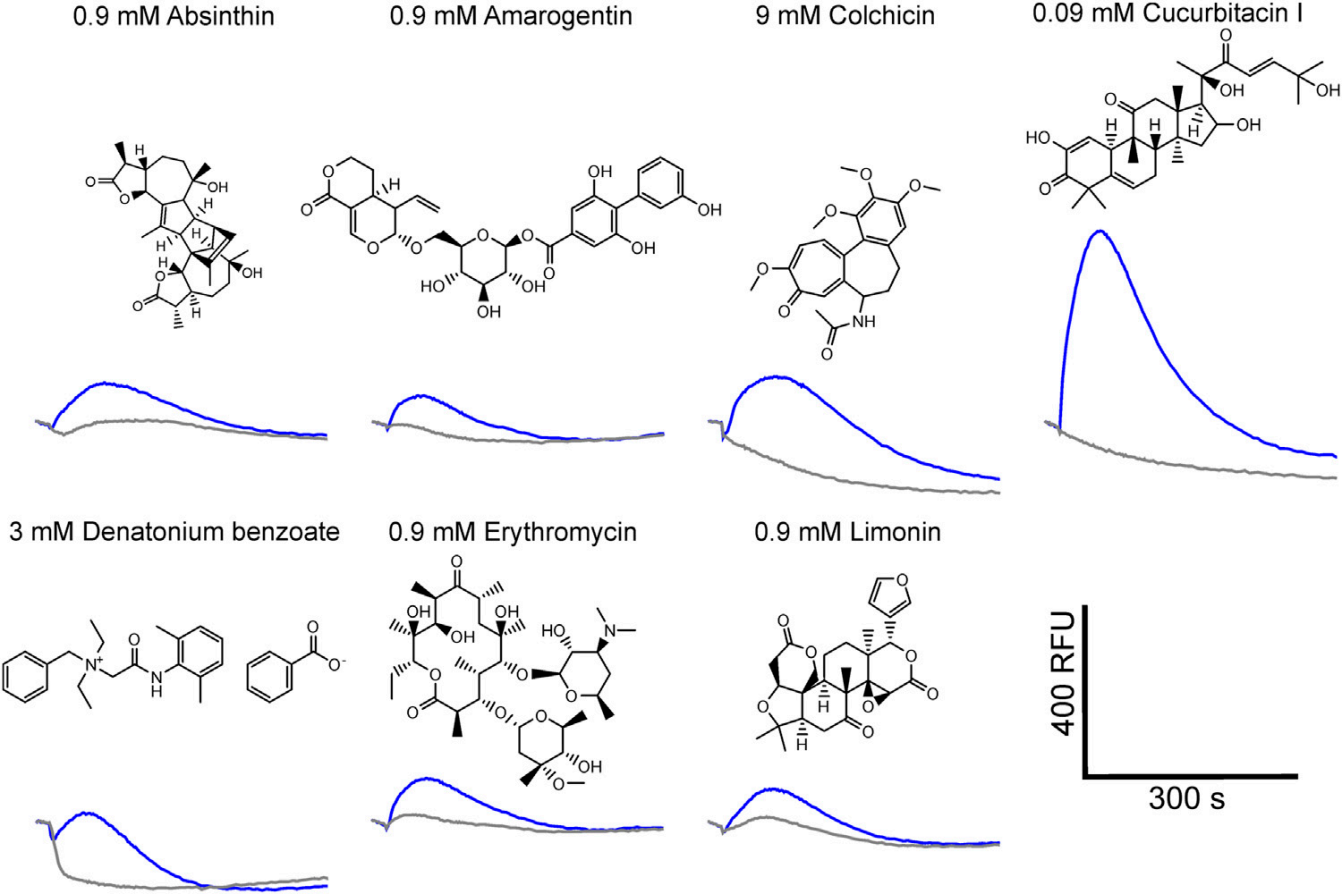
Fluorescence traces of tgTas2r1 expressing cells stimulated with bitter activators. The tgTas2r1 expression construct was transiently transfected in HEK 293T-Gα16gust44 cells and challenged with 72 compounds of a bitter compound library. The fluorescence traces of compounds eliciting responses (blue traces) are shown together with the corresponding substance concentrations. The traces were averaged from one representative experiment performed in duplicate wells. Empty vector transfected and identically treated cells were used as negative controls (gray traces). Scale bar, bottom right.

**FIGURE 4:**
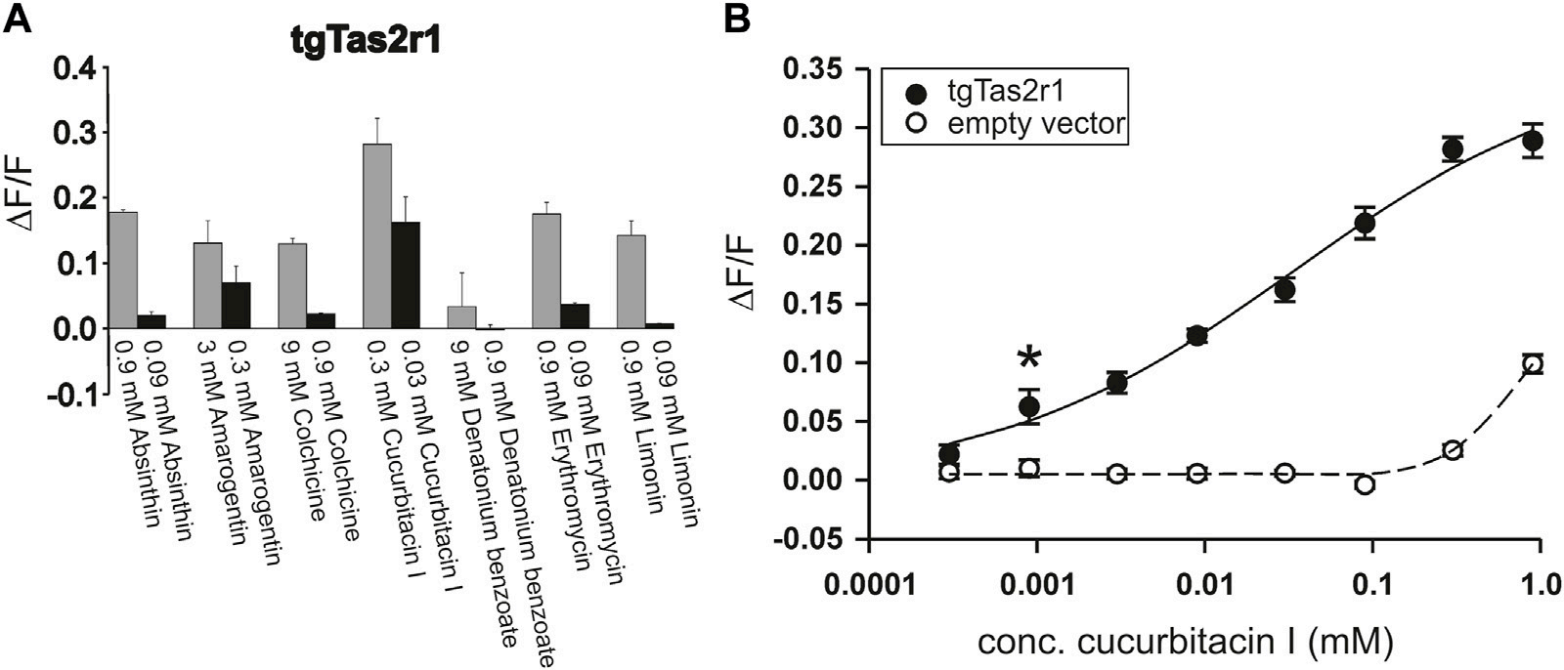
Functional screening of zebra finch bitter taste receptor tgTas2r1. tgTas2r1 was transiently transfected in HEK 293T-Gα16gust44 cells and screened with in total 72 natural and synthetic bitter compounds by calcium imaging. **(A)** Each compound resulting in the stimulation of a tgTas2r1 in a prescreening was tested in two different concentrations. The responses upon stimulation with the maximal concentration not leading to unspecific cellular responses, are shown by gray bars. The black bar representing lower concentration of compounds (1:10 dilution). The *y*-axis shows the relative fluorescence changes (∆F/F ± SD), and the *x*-axis is labeled with activating bitter compounds. **(B)** Dose-response relationship of tgTas2r1 with cucurbitacin I. The data are derived from two independent experiments each performed with 8 technical replicates for each concentration of receptor transfected and 4 corresponding technical replicates for empty vector transfected cells. The relative changes in fluorescence of receptor expressing cells (ΔF/F ± SEM, black circles, solid line) did not show clear signal saturation at concentrations (≤0.3 mM) with absent or tolerable receptor-independent artefact signals (*cf*. empty vector control, open circles, broken line). The asterisk indicates the threshold concentration defined as the lowest concentration at which receptor-transfected cells show a significant higher signal (*p* < 0.05) than empty vector transfected cells.

Using a functional heterologous expression assay, we screened tgTas2r1 with in total 72 substances of our bitter compound library. We observed receptor responses of tgTas2r1-expressing HEK 293T-Gα16gust44 cells with 7 bitter compounds (Figure 3). The newly identified 7 activators of the previously orphan receptor tgTas2r1 were further assessed for the relative fluorescence changes (ΔF/F) induced by the agonists at two different concentrations (Figure 4A).

The substance cucurbitacin I resulted in the highest signal amplitudes, whereas absinthin, amarogentin, colchicine, erythromycin and limonin resulted in considerably lower signal amplitudes. Denatonium benzoate activated tgTas2r1 only slightly. In order to investigate the cucurbitacin I responsiveness of tgTas2r1 in more detail, we monitored a full dose-response relationship (Figure 4B). Due to substantial receptor-independent artefacts at cucurbitacin I concentration above 0.3 mM (*cf*. plot of empty vector transfected cell signals), we were not able to deduce an EC_50_-concentration. The threshold concentration (defined as the lowest concentration of receptor expressing cells exhibiting statistically significant different fluorescence changes compared to identically treated empty vector transfected cells) was 0.0009 mM for cucurbitacin I.

The response rate of tgTas2r1 with 7 activators among the screened 72 substances (∼10%) suggests that this receptor belongs to the group with intermediate tuning breadths. The majority of compounds that were screened positive represent natural bitter compounds. Two of the natural new agonists, cucurbitacin I and colchicine, are quite powerful toxins targeting cytoskeletal structures of cells (Wang et al., 2017; Angelidis et al., 2018) which is in good agreement with the function of bitter taste receptors as warning sensors.

Interestingly, the agonist profile discovered for tgTas2r1 shows nearly no overlap with previously identified activators for tgTas2r5, -r6, and –r7, thus increasing the coverage of potentially harmful bitter substances in the habitat of zebra finch (Table 1). With four common agonists, tgTas2r1 exhibits the largest overlap with the agonist spectrum of chicken ggTas2r7, a very broadly tuned generalist receptor. A similar extent of agonist overlap is observed between tgTas2r1 and turkey mgTas2r3, although this agonist set is not identical to the overlap between tgTas2r1 and ggTas2r7.

**TABLE 1:** Activation of avian Tas2rs by bitter substances.

No.	Receptor substance	tgTas2r1	tgTas2r5	tgTas2r6	tgTas2r7	ggTas2r1	ggTas2r2	ggTas2r7	mgTas2r3	mgTas2r4
1	Absinthin	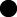						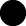		
2	Amarogentin	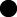						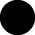	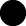	
3	Andrographolide			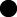	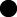			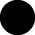	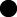	
4	Azathioprine					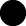				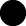
5	Caffeine						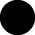			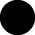
6	Camphor			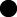	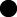					
7	Carisoprodol							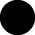	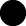	
8	Chloramphenicol		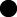			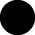	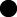	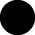	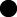	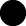
9	Chloroquine		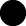			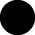				
10	Chlorpheniramine				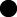	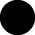	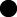	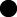		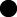
11	Colchicine	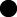						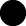	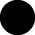	
12	Coumarin					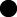	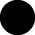			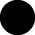
13	Cucurbitacin I	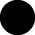								
14	Cycloheximide				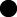			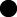		
15	Denatoniumbenzoate	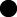	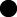		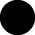					
16	Diphenhydramin					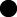				
17	Diphenidol		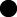	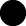		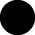	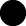	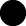	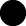	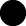
18	Erythromycin	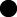						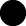	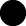	
19	Gingkolide A							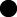	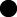	
20	Limonin	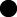							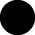	
21	Nicotin					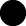				
22	Parthenolide						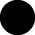	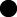	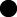	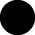
23	Picrotoxinin					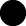		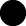	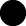	
24	PTC		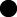							
25	Quassin							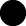		
26	Quinine sulphate					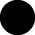	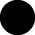	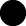	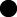	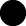
27	Saccharin								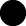	
28	(−)-α-Thujone							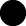	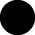	
29	Yohimbine						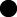	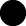	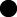	

The bitter compounds shown to activate zebra finch (tgTas2r), chicken (ggTas2r), and turkey (mgTas2r) bitter taste receptors are listed. The signal amplitudes are divided in 3 categories, weak (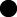, ΔF/F < 0.2), medium (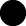, ΔF/F 0.2–0.4), and high (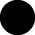, ΔF/F > 0.4).

## 3 Discussion

In the present manuscript, we characterized the activation profile of the zebra finch bitter taste receptor Tas2r1. Unlike the previously examined receptors Tas2r5, -r6, and –r7 that are orthologs of chicken Tas2r1 (Behrens et al., 2014), the zebra finch Tas2r1 belongs to a different branch of bird Tas2rs othologous to chicken Tas2r7 and turkey Tas2r3 (Figure 1). Our data show that tgTas2r1 clearly complements the bitter agonist spectrum of zebra finch (Table 1). Of the 7 substances identified, only a single, denatonium benzoate, is also activating other already investigated tgTas2rs. Of the 29 bitter activators summarized in table 1, 23 activate at least one chicken receptor, 19 activate turkey Tas2rs, and 15 activate zebra finch receptors. Hence, even though chicken and turkey possess fewer functional bitter taste receptors, they cover a broader bitter agonist spectrum. This may be due to zebra finch lacking representatives in the chicken Tas2r2/turkey Tas2r4 clade (Behrens et al., 2014) and (Figure 1), or the gap in the zebra finch bitter recognition spectrum might also be covered by the 3 tgTas2rs not investigated at present.

Despite its broad tuning for chemically diverse agonists, tgTas2r1 seems to be rather selective. Whereas the sesquiterpene lactone absinthin activates the receptor, the other sesquiterpene lactones included in the screening, parthenolide and picrotoxinin, exhibited no agonistic properties. Of the screened alkaloids, only colchicine induced responses. Moreover, all identified agonists belong to different chemical classes. The, by far, most pronounced response of tgTas2r1 was observed with the substance cucurbitacin I (Figures 3, 4). The apparent potency and efficacy of this ligand suggests an important role of this and related compounds in the ecology of zebra finch. Cucurbitacins are toxic compounds mostly found in plants of the cucurbitaceae family which include also well known edible varieties such as cucumber, zucchini and pumpkin (Kaushik et al., 2015). Birds can get in contact with cucurbitacins either directly by feeding on the plants or indirectly by insects, mostly beetles, sequestrating cucurbitacins as defense from predators (Rowell-Rahier et al., 1995; Gillespie et al., 2003). Although data on cucurbitacin sensitivity of birds are scarce, it was shown that European starlings, which belong to the same order of passerine birds like zebra finches, strongly avoid this compound class (Mason and Turpin, 1990). This observation is likely predictive also for an avoidance behavior of zebra finch, which has been shown to possess taste buds within the oral cavity (Heidweiller and Zweers, 1990). Another interesting observation made in chicken is the increase of bitter taste receptor gene expression by perinatal administration of bitter substances such as quinine (Cheled-Shoval et al., 2014). This may allow birds to adjust their bitter taste sensitivity to the occurrence of particular bitter plants in their individual habitats.

Depending on the plant, the plant part ingested and some seasonal variations, cucurbitacin levels can be considerable. For example, the plant *Trichosanthes cucumerina L*., used for traditional medicine in India, contains between 1.7 and 37 mg/kg of total curcurbitacines which equals roughly 3–70 μmol/kg (Attard et al., 2011). As we observed a threshold concentration as low as 0.9 µM with cucurbitacin I, it appears safe to assume that zebra finch are well equipped to recognize cucurbitacins at biologically relevant concentrations. The rather low cucurbitacin I LD_50_-concentration of 5 mg/kg body weight observed for mice (Gry et al., 2006) would translate to 1.6 g of the fruits containing the highest cucurbitacin concentration for zebra finch, which weigh only about 12 g. This suggests the necessity for sensitive aversive reactions in zebra finch.

In summary, our data confirm that, based on Tas2r function, birds do not exhibit inferior bitter taste abilities and that bitter taste perception, as in almost all other vertebrate species, fulfills a key role for their survival.

## Data Availability

The original contributions presented in the study are included in the article/Supplementary Material, further inquiries can be directed to the corresponding author.
